# Author Correction: TGF-β induces miR-100 and miR-125b but blocks let-7a through LIN28B controlling PDAC progression

**DOI:** 10.1038/s41467-019-11752-2

**Published:** 2019-08-14

**Authors:** Silvia Ottaviani, Justin Stebbing, Adam E. Frampton, Sladjana Zagorac, Jonathan Krell, Alexander de Giorgio, Sara M. Trabulo, Van T. M. Nguyen, Luca Magnani, Hugang Feng, Elisa Giovannetti, Niccola Funel, Thomas M. Gress, Long R. Jiao, Ylenia Lombardo, Nicholas R. Lemoine, Christopher Heeschen, Leandro Castellano

**Affiliations:** 1 Department of Surgery and Cancer, Division of Cancer, Imperial College London, Imperial Centre for Translational and Experimental Medicine (ICTEM), London, W12 0NN UK; 2 Department of Surgery and Cancer, HPB Surgical Unit, Imperial College, Hammersmith Hospital Campus, London, W12 0HS UK; 3 Department of Surgery and Cancer, Division of Cancer, Imperial College London, Institute of Reproductive and Developmental Biology (IRDB), London, W12 0NN UK; 4 Stem Cells & Cancer Group, Spanish National Cancer Research Centre (CNIO), Madrid, 28028 Spain; 5 Stem Cells in Cancer & Ageing, Barts Cancer Institute, Queen Mary University of London, London, EC1M 6BQ UK; 6 Epigenetics and Genome Stability Team, The Institute of Cancer Research, 237 Fulham Road, London, SW3 6JB UK; 7 Department of Medical Oncology, VU University Medical Center, Cancer Center Amsterdam, Amsterdam, 1081 HV The Netherlands; 8 Cancer Pharmacology Lab, AIRC Start-Up Unit, University of Pisa, Pisa, 56126 Italy; 9 Clinic for Gastroenterology, Endocrinology, Metabolism and Infectiology, Philipps-University Marburg, Marburg, 35037 Germany; 10 Centre for Molecular Oncology, Barts Cancer Institute, Queen Mary University of London, London, EC1M 6BQ UK; 11 University of Sussex, School of life Sciences, John Maynard Smith Building, Falmer, Brighton, BN1 9QG UK

**Keywords:** Cancer stem cells, Cell biology

Correction to: *Nature Communications* 10.1038/s41467-018-03962-x, published online 10 May 2018

The original version of this Article omitted a declaration from the competing interests statement, which should have included the following: ‘One of the authors, Y.L., is an editor on the staff of *Nature Communications*, but was not in any way involved in the journal review process. The other authors declare no competing interests’. This has now been corrected in both the PDF and HTML versions of the Article.

