# Supplementary material for: TGF-β induces miR-100 and miR-125b but blocks let-7a through LIN28B controlling PDAC progression
Source: Nat Commun. 2018 May 10;9:1845. doi: 10.1038/s41467-018-03962-x (PMC5945639; doi:10.1038/s41467-018-03962-x)
Supplement: Supplementary file 1 — Supplementary Information [file 41467_2018_3962_MOESM1_ESM.pdf]

TGF -ß induces miR-100 and miR-125b but blocks let-7a through LIN28B controlling PDAC progression

Ottaviani et al.

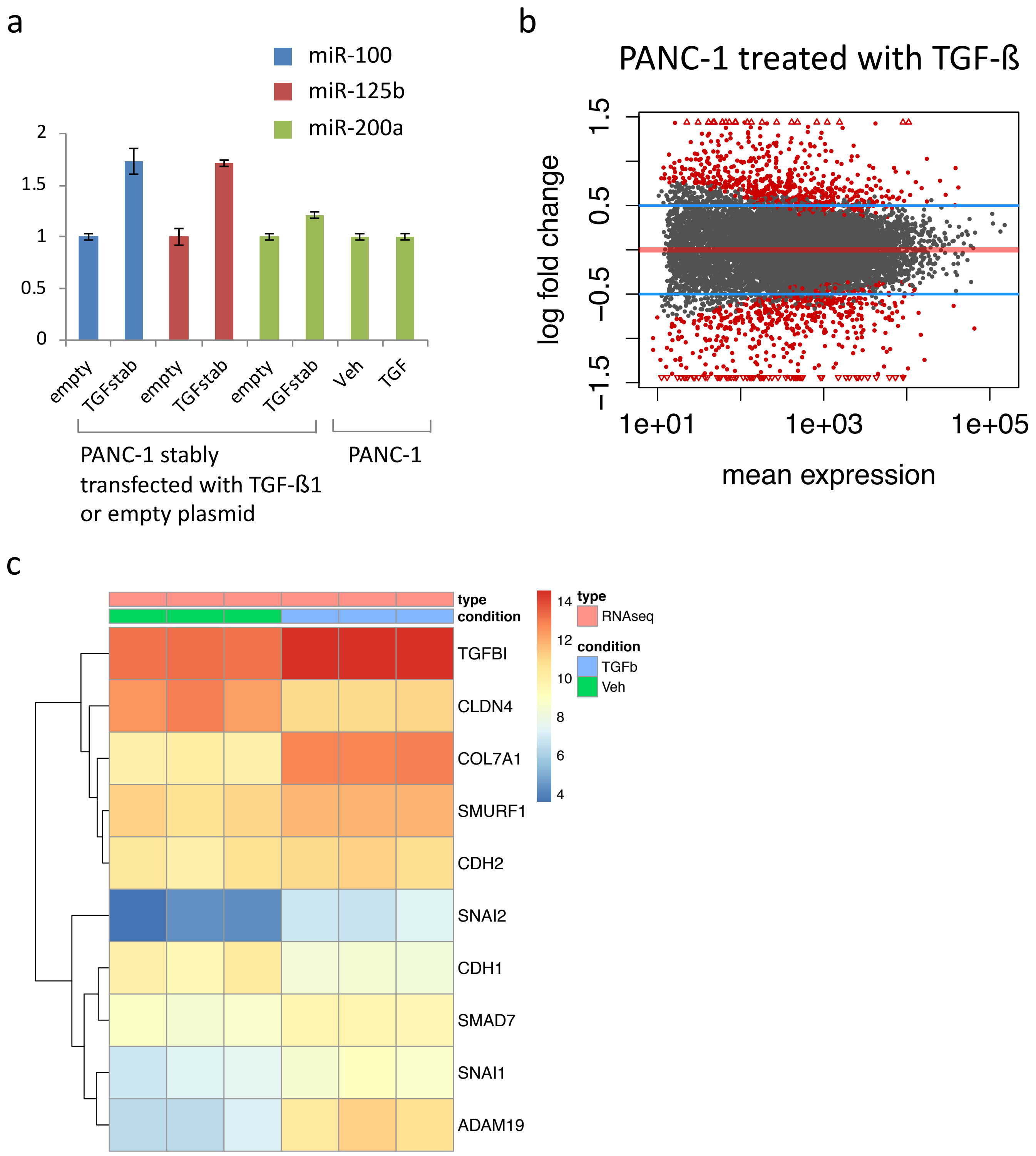

Supplementary Figure 1. TGF-ß-dependent EMT signature in PANC-1 cells. (**a**) RT-qPCR for miR-100, miR-125b or miR-200a in PANC-1 cells stably over-expressing TGF-ß1 (TGFstab) or control line (empty) or PANC-1 treated with TGF-ß for 72h. Values were normalized to RNU47 levels and are shown as mean ± s.e.m. Results are from three independent experiments each performed in triplicate. (**b**) MA-plot of gene expression change obtained from RNA-seq data of PANC-1 cells treated with vehicle or TGF-ß for 72h analysed by DESeq2 (*n*=3 for each condition). Significantly up and down-regulated genes are shown in red. (**c**) Heatmap from RNA-seq data showing up- and down-regulation of a subset of EMT regulating genes.

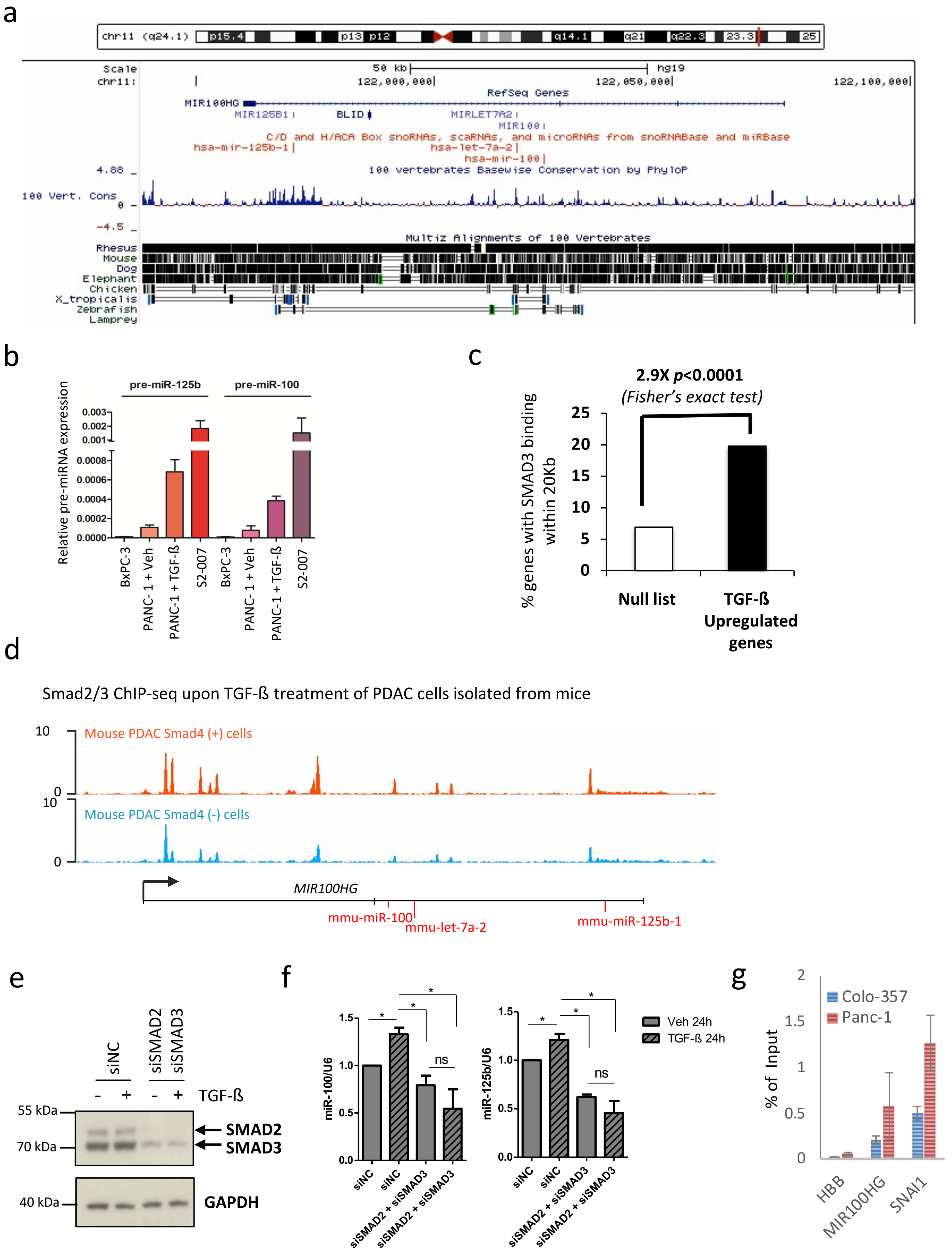

a
chr11 (q24.1)
Scale
chr11:
50 kb
hg19
122,000,000
122,050,000
122,100,000
RefSeq Genes
MIR100HG
MIR125B1
BLID
MIRLET7A2
MIR100
C/D and H/ACA Box snoRNAs, scaRNAs, and microRNAs from snoRNABase and miRBase
hsa-mir-125b-1
hsa-let-7a-2
hsa-mir-100
100 vertebrates Basewise Conservation by PhyloP
100 Vert. Cons
Multiz Alignments of 100 Vertebrates
Rhesus
Mouse
Dog
Elephant
Chicken
X_tropicalis
Zebrafish
Lamprey
b
pre-miR-125b
pre-miR-100
Relative pre-miRNA expression
BxPC-3
PANC-1 + Veh
PANC-1 + TGF-ß
S2-007
c
2.9X p<0.0001
(Fisher's exact test)
% genes with SMAD3 binding within 20Kb
Null list
TGF-ß Upregulated genes
d
Smad2/3 ChIP-seq upon TGF-ß treatment of PDAC cells isolated from mice
Mouse PDAC Smad4 (+) cells
Mouse PDAC Smad4 (-) cells
MIR100HG
mmu-miR-100
mmu-let-7a-2
mmu-miR-125b-1
e
siNC
siSMAD2
siSMAD3
TGF-ß
55 kDa
70 kDa
40 kDa
SMAD2
SMAD3
GAPDH
f
miR-100/U6
miR-125b/U6
ns
siNC
siSMAD2 + siSMAD3
Veh 24h
TGF-ß 24h
g
Colo-357
Panc-1
% of Input
HBB
MIR100HG
SNAI1

Supplementary Figure 2. TGF-ß induces miR-100 and miR-125b through SMAD2/3 transcription factors. (**a**) UCSC genome browser snapshot (http://genome.ucsc.edu/; hg19) showing MIR100HG genomic locus, RefSeq annotation, miRNAs position and level of conservation. (**b**) RT-qPCR for precursor miR-125b (pre-miR-125b) or precursor miR-100 (pre-miR-100) in PDAC cell lines. Values were normalized to U6 levels. (**c**) Significant enrichment of ChIP-seq SMAD2/3 peaks within 20 kilobases from the transcription start sites of TGF-ß up-regulated genes. *P* values were calculated using Fisher's exact test. (**d**) SMAD2/3 ChIP-seq binding to the *MIR100HG* locus in mouse PDAC cells. (**e**) Immunoblot for SMAD2/3 and GAPDH (endogenous control) from PANC-1 cells treated with negative control siRNA (siNC) or siRNA for SMAD2 (siSMAD2) in combination with siRNA for SMAD3 (siSMAD3) for 48 hours. At 24 hours post siRNA transfection TGF-ß or vehicle were added for 24 hours. (**f**) RT-qPCR of miR-100 (*left*) or miR-125b (*right*) from PANC-1 cells treated as in **e**. Values were normalized to U6 levels. (**g**) ChIP-qPCR analysis of SMAD2/3 binding at miR100HG and SNAI1 promoter regions in COLO357 and PANC-1 cells treated with TGF-ß. HBB was used as negative control. In **b**,**f**,**g** values are shown as mean ± s.e.m. Results are from three independent experiments each performed in triplicate. *P-value < 0.05. P values were calculated using two-tailed Student's t test.

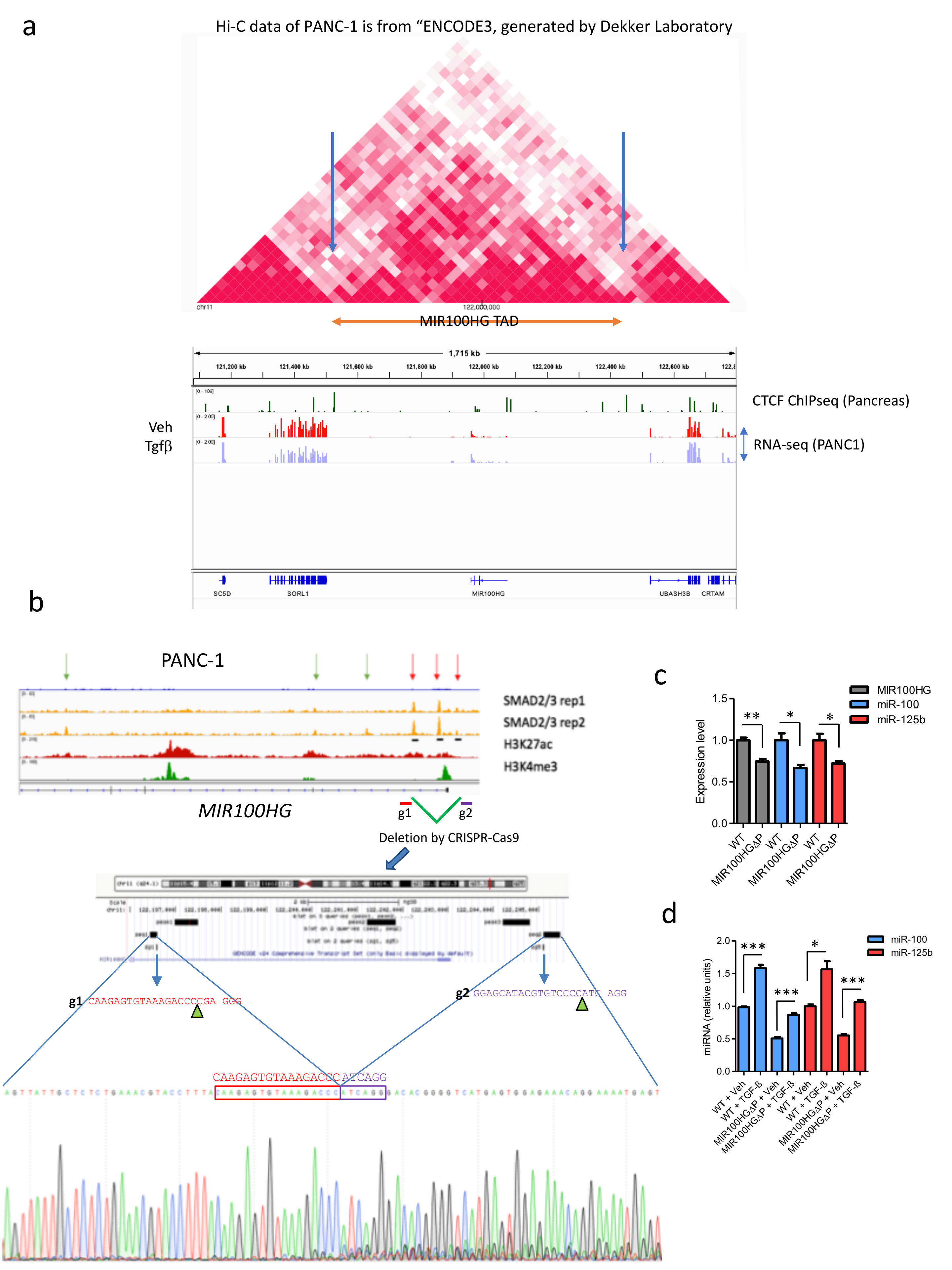
a
Hi-C data of PANC-1 is from "ENCODE3, generated by Dekker Laboratory
chr11
122,000,000
MIR100HG TAD
1,715 kb
121,200 kb
121,400 kb
121,600 kb
121,800 kb
122,000 kb
122,200 kb
122,400 kb
122,600 kb
CTCF ChIPseq (Pancreas)
Veh
Tgfβ
RNA-seq (PANC1)
SC5D
SORL1
MIR100HG
UBASH3B
CRTAM
b
PANC-1
SMAD2/3 rep1
SMAD2/3 rep2
H3K27ac
H3K4me3
MIR100HG
g1
g2
Deletion by CRISPR-Cas9
g1 CAAGAGTGTAAAGACCCCGA GGG
g2 GGAGCATACGTGTCCCCATC AGG
CAAGAGTGTAAAGACCC ATCAGG
c
MIR100HG
miR-100
miR-125b
Expression level
0.0
0.5
1.0
1.5
**
*
*
WT
MIR100HGΔP
WT
MIR100HGΔP
WT
MIR100HGΔP
d
miR-100
miR-125b
miRNA (relative units)
0.0
0.5
1.0
1.5
2.0
***
***
*
***
WT + Veh
WT + TGF-ß
MIR100HGΔP + Veh
MIR100HGΔP + TGF-ß
WT + Veh
WT + TGF-ß
MIR100HGΔP + Veh
MIR100HGΔP + TGF-ß

Supplementary Figure 3. Genomic deletion of SMAD2/3 interaction sites within MIR100HG promoter. (**a**) Hi-C data of PANC-1 comes from ENCODE. Analyses and graphs were performed using online tools from http://promoter.bx.psu.edu/hi-c/**.** (**b**) Schematic of the strategy for deletion of SMAD2/3 interacting sites by CRISPR-Cas9 in PANC-1 cells. Validation of the deletion is shown by the sequencing chromatogram at the bottom. (**c**) RT-qPCR of MIR100HG (normalized to GAPDH levels), miR-100 and miR-125b (normalized to RNU44 levels) in PANC-1 wild-type cells (WT) or in CRISPR cells edited for deletion of SMAD2/3 interacting sites in the promoter region of MIR100HG (MIR100HGΔP). (**d**) RT-qPCR of miR-100 and miR-125b in cells described in **c**. Cells were treated with vehicle (Veh) or TGF-ß for 72 hours. In **c**,**d** values represent the mean ± s.e.m. Results are from three independent experiments each performed in triplicate. **P*-value < 0.05, ***P*-value < 0.01, ****P*-value < 0.001. *P* values were calculated using two-tailed Student's *t* test.

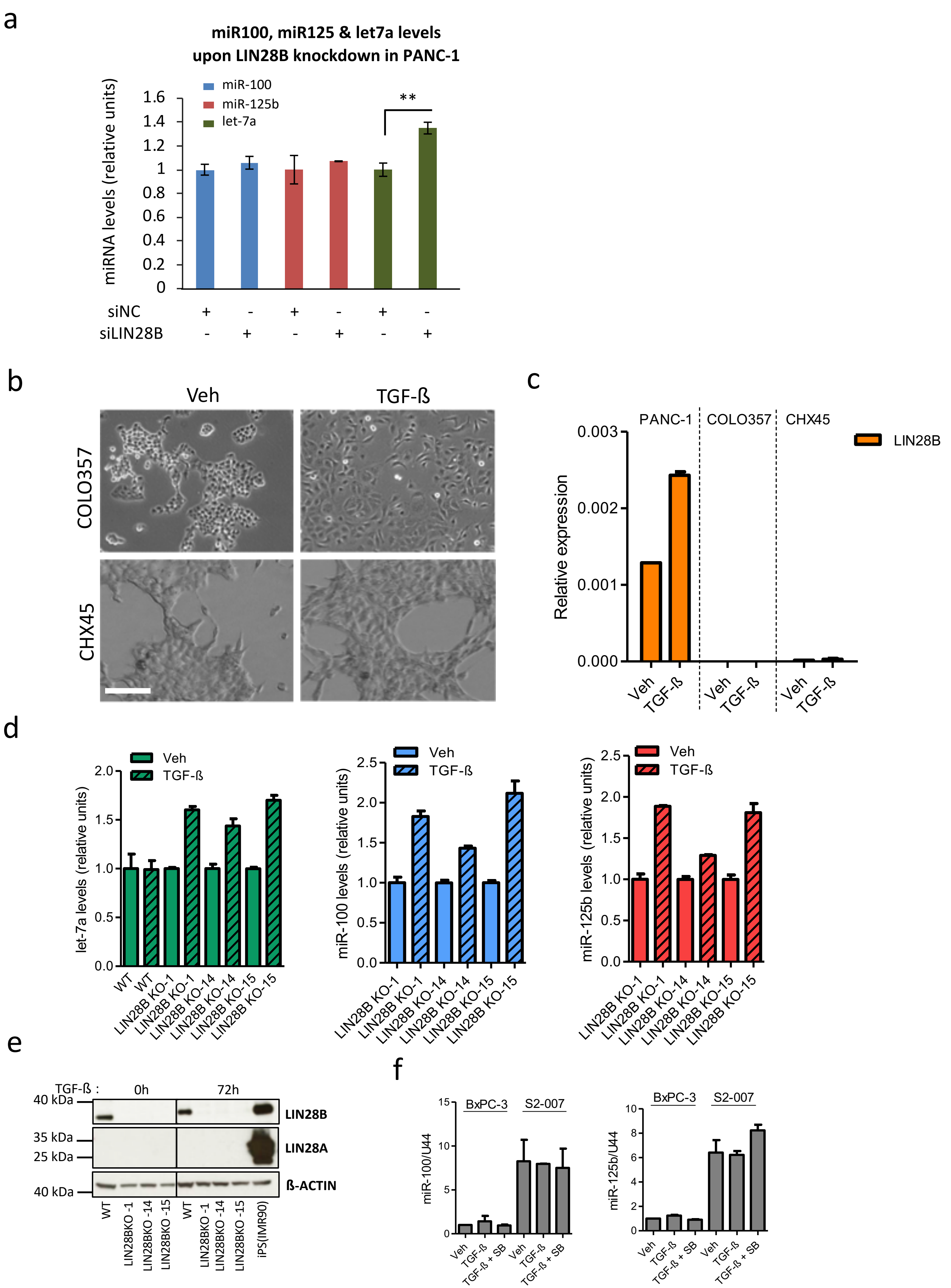
a
miR100, miR125 & let7a levels
upon LIN28B knockdown in PANC-1
miR-100
miR-125b
let-7a
**
miRNA levels (relative units)
1.6
1.4
1.2
1
0.8
0.6
0.4
0.2
0
siNC
siLIN28B
b
Veh
TGF-ß
COLO357
CHX45
c
PANC-1
COLO357
CHX45
LIN28B
Relative expression
0.003
0.002
0.001
0.000
Veh
TGF-ß
d
Veh
TGF-ß
let-7a levels (relative units)
miR-100 levels (relative units)
miR-125b levels (relative units)
WT
LIN28B KO-1
LIN28B KO-14
LIN28B KO-15
e
TGF-ß :
0h
72h
40 kDa
35 kDa
25 kDa
LIN28B
LIN28A
ß-ACTIN
LIN28BKO -1
LIN28BKO -14
LIN28BKO -15
iPS(IMR90)
f
BxPC-3
S2-007
miR-100/U44
miR-125b/U44
TGF-ß + SB

Supplementary Figure 4. TGF-ß induces miR-100 and miR-125b but not let-7a by co-regulation of MIR100HG and LIN28B. (**a**) RT-qPCRs of miR-100, miR-125b and let-7a in PANC-1 cells treated with negative control siRNA (siNC) or siRNA for LIN28B (siLIN28B). Results were normalized to RNU44 levels. (**b**) Representative phase-contrast images of COLO357 and CHX45 cells treated with vehicle (Veh) or TGF-ß for 72 hours. Scale bar: 100µm. (**c**) Bar-chart showing levels of LIN28B transcripts, measured by RT-qPCR in PANC-1, COLO357 and CHX45 cells, treated with vehicle (Veh) or with TGF-ß for 72 hours. Results were normalized to GAPDH levels. (**d**) RT-qPCR expression levels of let7a (*left*), miR-100 (*center*) or miR-125b (*right*) in PANC-1 wild-type cells (WT) or in three independent LIN28B KO clones (*n*=3) generated by CRISPR-Cas9 (LIN28B KO-1, LIN28B KO-14, LIN28B KO-15) treated with vehicle (Veh) or TGF-ß for 72 hours. Results were normalized to RNU44 levels. (**e**) Immunoblots of LIN28B, LIN28A or ß-ACTIN (endogenous control) in cells as in **d**. iPS IMR90 were used as positive control for the expression of LIN28A. (**f**) RT-qPCR of miR-100 (*left*) or miR-125b (*right*) in BxPC-3 or S2-007 cells treated with vehicle (Veh) or TGF-ß (5nM) or TGF-ß in combination with SB431542 (SB; 2.5 µg/ml) for 24 hours. Results were normalized to RNU44 levels. In **a,c,d** and **f** values represent the mean ± s.e.m. Results are from three independent experiments each performed in triplicate. ***P*-value < 0.01. *P* values were calculated using two-tailed Student's *t* test.

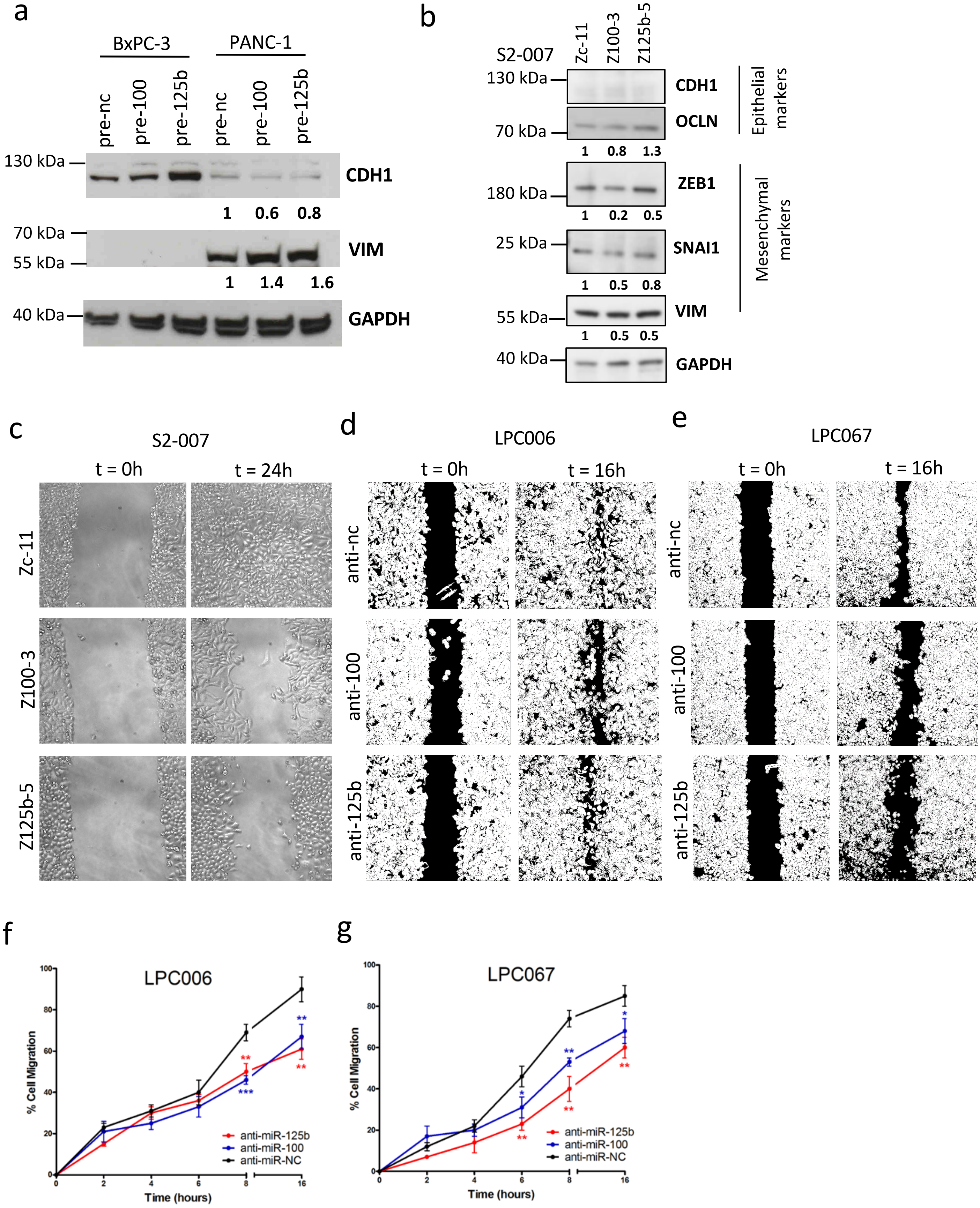
a
BxPC-3
PANC-1
pre-nc
pre-100
pre-125b
130 kDa
CDH1
1 0.6 0.8
70 kDa
55 kDa
VIM
1 1.4 1.6
40 kDa
GAPDH
b
S2-007
Zc-11
Z100-3
Z125b-5
130 kDa
CDH1
OCLN
70 kDa
1 0.8 1.3
Epithelial markers
ZEB1
180 kDa
1 0.2 0.5
25 kDa
SNAI1
1 0.5 0.8
Mesenchymal markers
55 kDa
VIM
1 0.5 0.5
40 kDa
GAPDH
c
S2-007
t = 0h
t = 24h
Zc-11
Z100-3
Z125b-5
d
LPC006
t = 0h
t = 16h
anti-nc
anti-100
anti-125b
e
LPC067
t = 0h
t = 16h
anti-nc
anti-100
anti-125b
f
LPC006
% Cell Migration
Time (hours)
anti-miR-125b
anti-miR-100
anti-miR-NC
g
LPC067
% Cell Migration
Time (hours)
anti-miR-125b
anti-miR-100
anti-miR-NC

Supplementary Figure 5. miR-100 and miR-125b promote EMT and motility. (**a**) Immunoblot for EMT markers E-cadherin (CDH1) or vimentin (VIM) in BxPC-3 or PANC-1 cells transfected with control mimic (pre-nc) or mature miRNA mimics (pre-miRs). GAPDH was used as loading control. Quantifications were calculated by measuring the intensity of the protein of interest/GAPDH. Representative images from three independent experiments are shown. (**b**) Immunoblot for EMT markers (CDH1, OCLN, ZEB1, SNAI1, VIM) in S2-007 Zip clones for miR-100 (Z100-3) miR-125 (Z125b-5) or control (Zc-11). GAPDH was used as loading control. Quantifications were calculated by measuring the intensity of the protein of interest/GAPDH (**c**) Time-lapse microscopy of wound-healing migration assay using S2-007 Zip clones as in **b** (See also Supplementary Movies 1-3). Representative pictures from three independent experiments are shown here. (**d-e**) Wound-healing migration assay in LPC006 in **d** and LPC067 cells in **e** transfected with anti-miR-NC, anti-miR-100 or anti-miR-125b (100nM). (**f-g**) Quantification of **d** and **e**. Values represent the mean ± s.d. Results are from three independent experiments each performed in triplicate. **P*-value < 0.05, ***P*-value < 0.01, ****P*-value < 0.001. *P* values were calculated using two-tailed Student's *t* test.

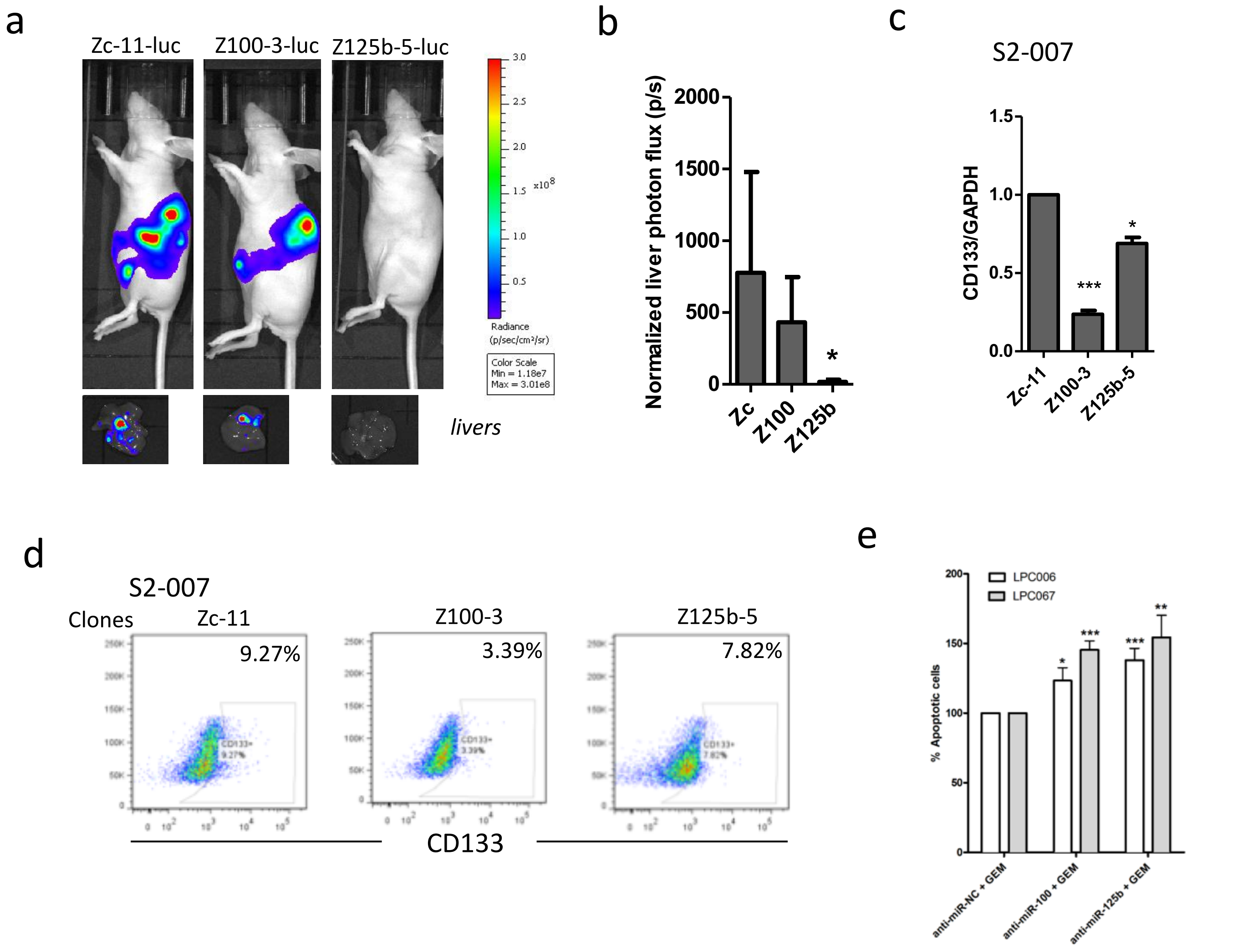

Supplementary Figure 6. Effects of miR-100 and miR-125b on metastasis of xenografted mouse models and apoptosis of GEM-treated PDAC primary cells. (**a**) 25x10^4 S2-007 Zc-11, Z100-3 or Z125b-5 all stably overexpressing luciferase (luc) were injected into the spleen. The spleen was removed 7 days post-injection and liver colonization was assessed by non-invasive bioluminescence imaging (BLI) at day 22 after injection. S2-007 cell colonization in the liver was further confirmed by BLI assessment in the livers (bottom) (*n*=6 mice per group). (**b**) Quantification of liver colonization. BLI signal at day 22 was normalized to signal at day 1 post-injection. **P*-value < 0.05. *P* values were calculated using one-sided Mann-Whitney test. (**c**) RT-qPCR of CD133 levels in S2-007 Zip clones. Values were normalized to GAPDH levels and are shown as mean ± s.e.m. Results are from three independent experiments each performed in triplicate. (**d**) CD133 protein level analysed by FACS in S2-007 Zip clones. (**e**) Apoptosis analysis of LPC006 and LPC067 cells transfected with anti-miR-NC, anti-miR-100 or anti-miR-125b (100nM) for 24h following treatment with gemcitabine (GEM; 1µM) for 24 hours. Data are shown as mean ± s.d. Results are from three independent experiments each performed in triplicate. **P*-value < 0.05, ***P*-value < 0.01, *** *P*-value < 0.001. *P* values were calculated using two-tailed Student's *t* test.

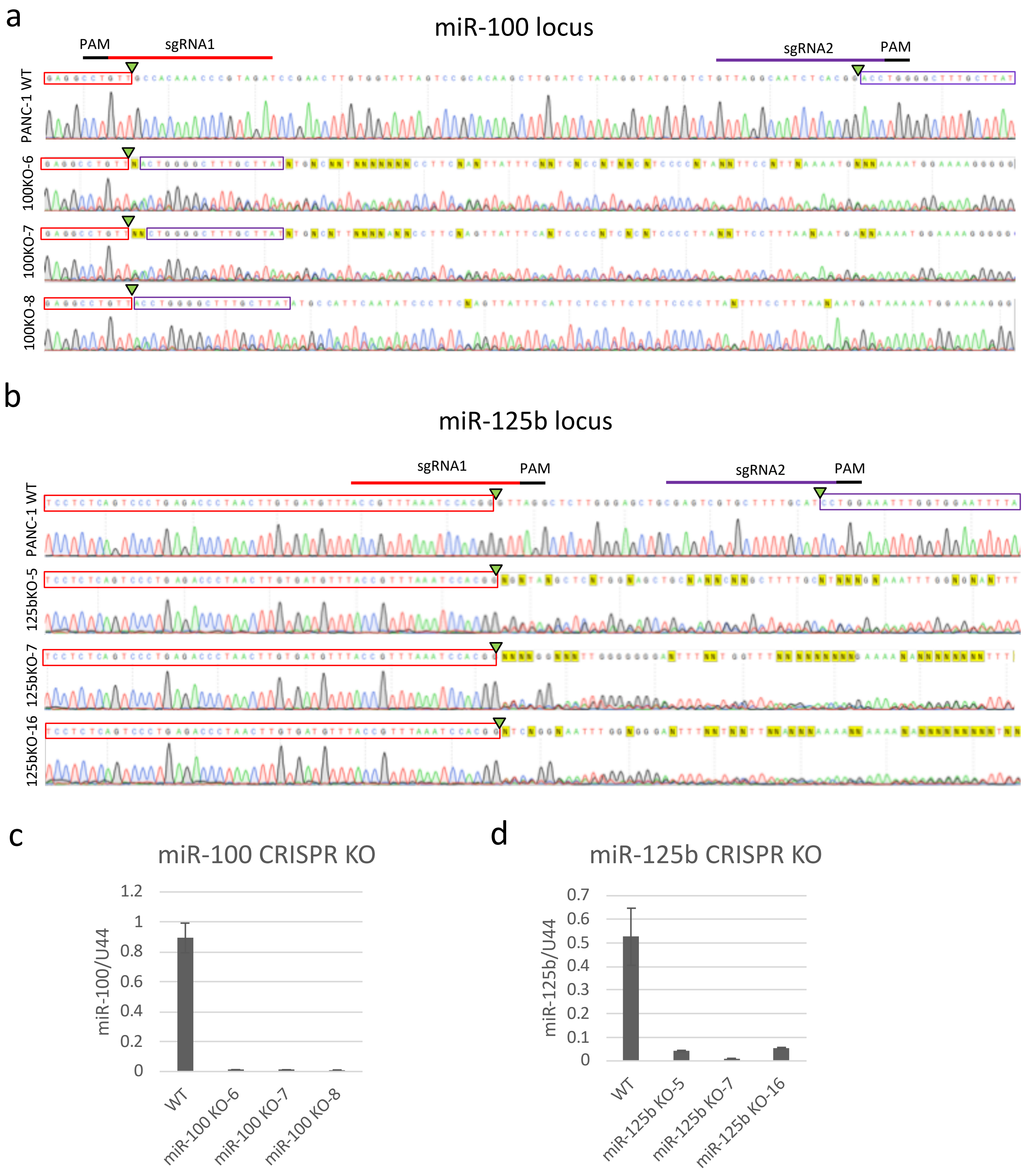

Supplementary Figure 7. Validation of miR-100 and miR-125b KO clones generated by CRISPR-Cas9. (**a**) Sequencing chromatograms of the miR-100 genomic locus in PANC-1 wild-type cells (WT) and three independent CRISPR KO clones for miR-100 (KO-6, KO-7, KO-8). (**b**) Sequencing chromatograms of the miR-125b genomic locus in PANC-1 WT cells and three independent CRISPR KO clones for miR-125b (KO-5, KO-7, KO-16). (**c**) RT-qPCR of miR-100 in WT and miR-100 KO clones (**d**) RT-qPCR of miR-125b in WT and miR-125b KO clones. Values were normalized to RNU47 levels and are shown as mean ± s.e.m. Results are from three independent experiments each performed in triplicate.

a

PANC-1

Cell line	# tumours/ # injections
Zc-1-TFG-ß1	3/5
Zc-1-empty	1/5
Z100-23-TGF-ß1	2/5
Z125b-6-TGF-ß1	0/5

Tumour take after 5 weeks

b

c

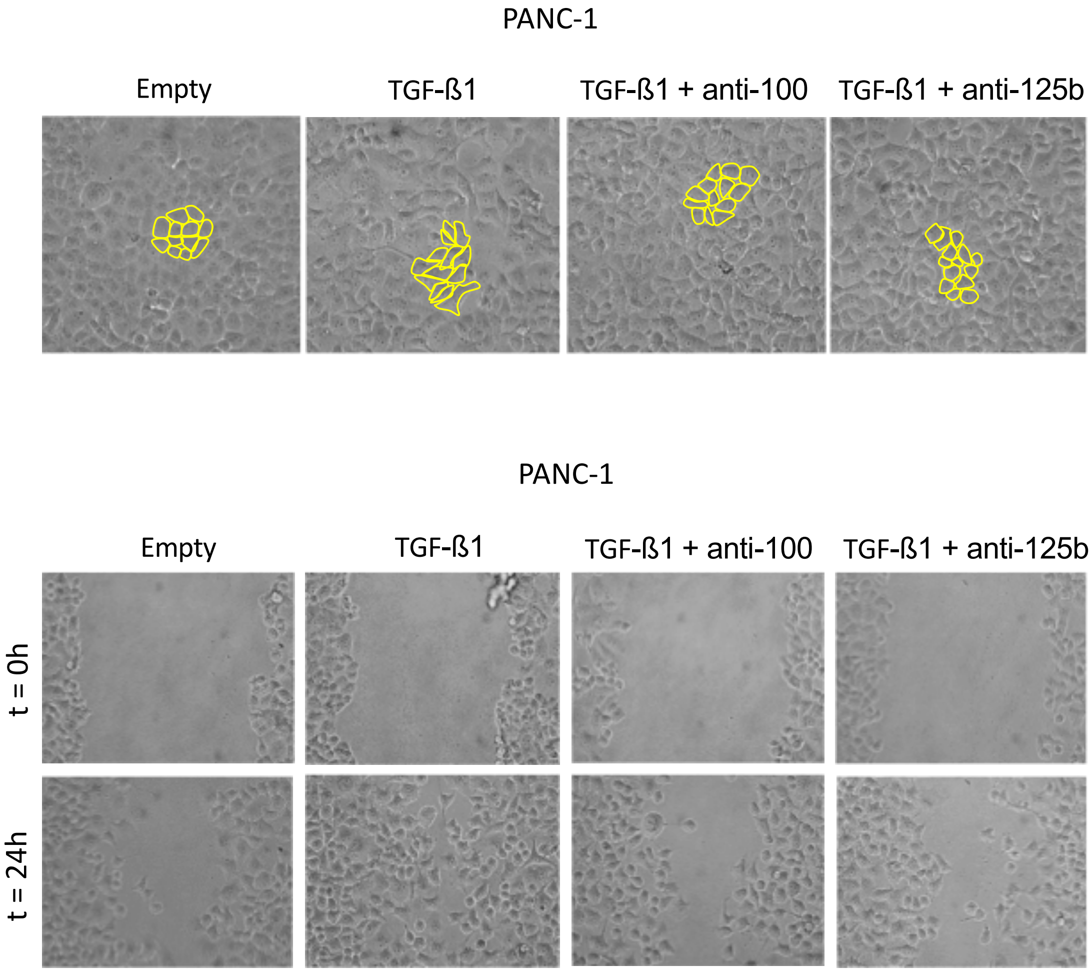

Supplementary Figure 8. miR-100 and miR-125b reduce TGF-ß-induced spindle-shaped cells and motility, but only miR-125b impairs TGF-ß-mediated tumorigenesis *in vivo*. (**a**) Two million PANC-1 Zip clones (Z100-23, Z125b-6 or Zc-1) all stably expressing TGF-ß1 or empty vector for Zc-11, were injected in both flanks of nude mice and tumour take was determined 5 weeks post-injection (*n*=5 per group). (**b**) PANC-1 cells stably overexpressing TGF-ß1 (TGF-ß1) or empty vector (Empty) were transiently transfected with the indicated anti-miRNA inhibitors for 48 hours and cellular shape was manually delineated. (**c**) Wound-healing migration assay in cells treated as in **b**. Representative images from three independent experiments are shown here.

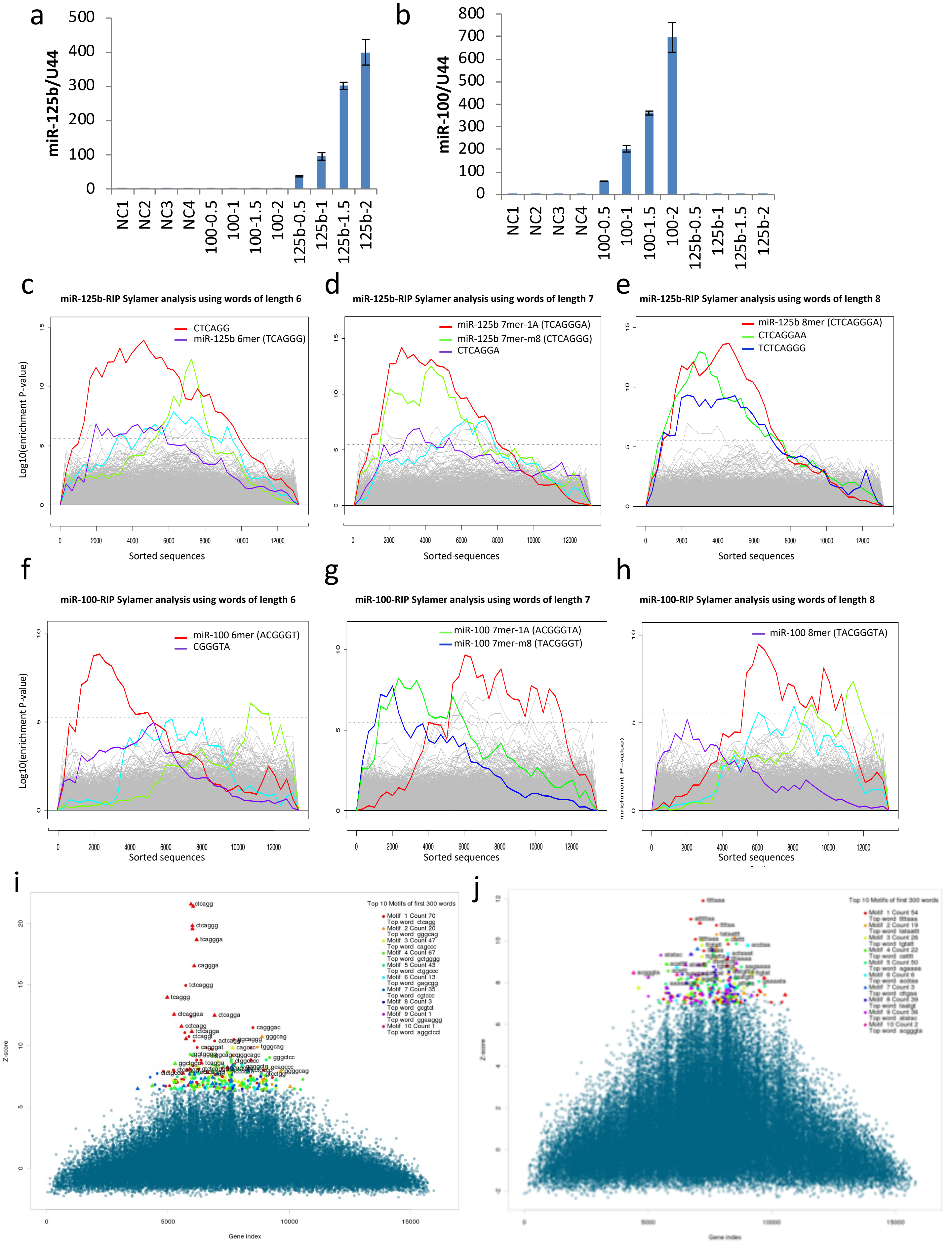
a
miR-125b/U44
NC1
NC2
NC3
NC4
100-0.5
100-1
100-1.5
100-2
125b-0.5
125b-1
125b-1.5
125b-2
b
miR-100/U44
c
miR-125b-RIP Sylamer analysis using words of length 6
CTCAGG
miR-125b 6mer (TCAGGG)
Log10(enrichment P-value)
Sorted sequences
d
miR-125b-RIP Sylamer analysis using words of length 7
miR-125b 7mer-1A (TCAGGGA)
miR-125b 7mer-m8 (CTCAGGG)
CTCAGGA
Sorted sequences
e
miR-125b-RIP Sylamer analysis using words of length 8
miR-125b 8mer (CTCAGGGA)
CTCAGGAA
TCTCAGGG
Sorted sequences
f
miR-100-RIP Sylamer analysis using words of length 6
miR-100 6mer (ACGGGT)
CGGGTA
Log10(enrichment P-value)
Sorted sequences
g
miR-100-RIP Sylamer analysis using words of length 7
miR-100 7mer-1A (ACGGGTA)
miR-100 7mer-m8 (TACGGGT)
Sorted sequences
h
miR-100-RIP Sylamer analysis using words of length 8
miR-100 8mer (TACGGGTA)
Sorted sequences
i
Top 10 Motifs of first 300 words
Motif 1 Count 70
Top word ctcagg
Motif 2 Count 20
Top word gggcag
Motif 3 Count 47
Top word cagccc
Motif 4 Count 67
Top word gctgggg
Motif 5 Count 43
Top word ctggccc
Motif 6 Count 13
Top word gagcgg
Motif 7 Count 35
Top word cgtccc
Motif 8 Count 3
Top word gcgtct
Motif 9 Count 1
Top word ggaaggg
Motif 10 Count 1
Top word aggctcct
Z-score
Gene index
j
Top 10 Motifs of first 300 words
Gene index

Supplementary Figure 9. Seed-enrichment analyses of miR-100 and miR-125b regulate gene targets. (**a-b**) RT-qPCR for miR-100 in **a** or miR-125b in **b** in PANC-1 cells treated with increasing concentration (0.5-2nM) of precursor miRNA mimics for 24h. Values were normalized to RNU44 levels and are shown as mean ± s.e.m. Results are from three independent experiments each performed in triplicate. (**c-e**) Sylamer enrichment plots for 6-8mers present in 3′UTR of transcripts ordered from the most to the least enriched onto AGO2 after overexpression of miR-125b in PANC-1. (**f-h**) Sylamer enrichment plots for 6-8mers present in 3′UTR of transcripts ordered from the most to the least enriched onto AGO2 after overexpression of miR-100 in PANC-1. (**i**) cWord enrichment plots for 6-8mers present in 3′UTR of transcripts ordered from the most to the least enriched onto AGO2 after overexpression of miR-125b in PANC-1. (**j**) cWord enrichment plots for 6-8mers present in 3′UTR of transcripts ordered from the most to the least enriched onto AGO2 after overexpression of miR-100 in PANC-1.

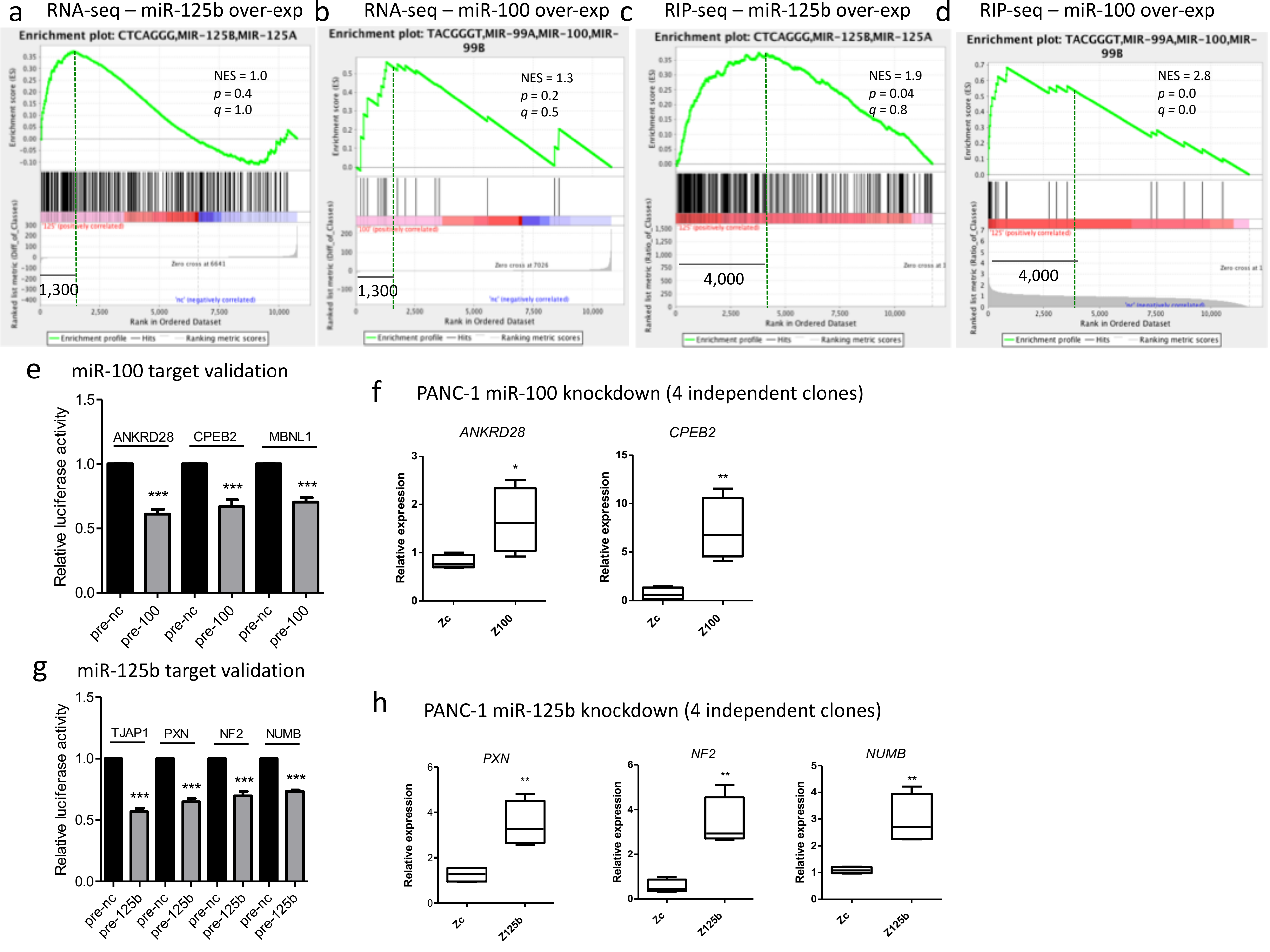

a RNA-seq – miR-125b over-exp
Enrichment plot: CTCAGGG,MIR-125B,MIR-125A
NES = 1.0
p = 0.4
q = 1.0
1,300
b RNA-seq – miR-100 over-exp
Enrichment plot: TACGGGT,MIR-99A,MIR-100,MIR-99B
NES = 1.3
p = 0.2
q = 0.5
1,300
c RIP-seq – miR-125b over-exp
Enrichment plot: CTCAGGG,MIR-125B,MIR-125A
NES = 1.9
p = 0.04
q = 0.8
4,000
d RIP-seq – miR-100 over-exp
Enrichment plot: TACGGGT,MIR-99A,MIR-100,MIR-99B
NES = 2.8
p = 0.0
q = 0.0
4,000
Rank in Ordered Dataset
Enrichment profile
Hits
Ranking metric scores
e miR-100 target validation
Relative luciferase activity
ANKRD28
CPEB2
MBNL1
pre-nc
pre-100
f PANC-1 miR-100 knockdown (4 independent clones)
ANKRD28
CPEB2
Relative expression
Zc
Z100
g miR-125b target validation
Relative luciferase activity
TJAP1
PXN
NF2
NUMB
pre-nc
pre-125b
h PANC-1 miR-125b knockdown (4 independent clones)
PXN
NF2
NUMB
Relative expression
Zc
Z125b

Supplementary Figure 10. miR-100 or miR-125b regulated targets. (**a-b**) Gene set enrichment analysis (GSEA) plots showing enrichment for miR-125b 7mer-m8 interacting motif (CTCAGGG) in **a** or miR-100 7mer-m8 interacting motif (TACGGGT) in **b** from RNA-seq gene list sorted in an ascending order starting from the most down-regulated one, according to fold-change in miR-125b or miR-100 overexpression respectively versus negative control. The top 1,300 genes were considered for further analyses. (**c-d**) GSEA plot showing enrichment for miR-125b 7mer-m8 in **c** or miR-100 7mer-m8 in **d** from RIP-seq gene list sorted in a descending order from the most enriched transcript onto AGO2 after miR-125b or miR-100 overexpression versus negative control. The top 4,000 genes were considered for further analyses. Details are listed in **Method** section. (**e**) Validation of a subset of miR-100 targets by luciferase reporter assay. The targets were chosen from the overlap gene list (overlap between the top 4000 transcripts most enriched in RIP-seq and top 1300 downregulated transcripts in RNA-seq. See also **Figure 7b**). PANC-1 were co-transfected with control mimic (pre-nc) or precursor miR-100 (pre-100) together with the indicated UTR reporter constructs. Luciferase activity was measured 24h post-transfection. Data are shown as mean ± s.e.m. Results are from three independent experiments each performed in triplicate. (**f**) Validation of a subset of miR-100 targets by RT-qPCR in four independent PANC-1 Zip clones (*n*=4). (Zc include Zc-1, Zc-3, Zc-7, Zc-8; Z100 include Z100-23, Z100-29, Z100-48, Z100-48). (**g**) Validation of a subset of miR-125b targets by luciferase reporter assay. The targets were chosen from the overlap gene list (**See Figure 7a**). PANC-1 were co-transfected with control mimic (pre-nc) or precursor miR-125b (pre-125b) together with the indicated UTR reporter constructs. Luciferase activity was measured as in **e**. (**h**) Validation of a subset of miR-125b targets by RT-qPCR in four independent PANC-1 Zip clones (*n*=4). (Control same as in **f**; Z125b include Z125b-6, Z125b-15, Z125b-17, Z125b-26) In **f, h** Box plots show median and whiskers are minimum and maximum. Results are from three independent experiments each performed in triplicate. **P*-value < 0.05, ***P*-value < 0.01, *** *P*-value < 0.001. *P* values were calculated using two-tailed Student's *t* test.

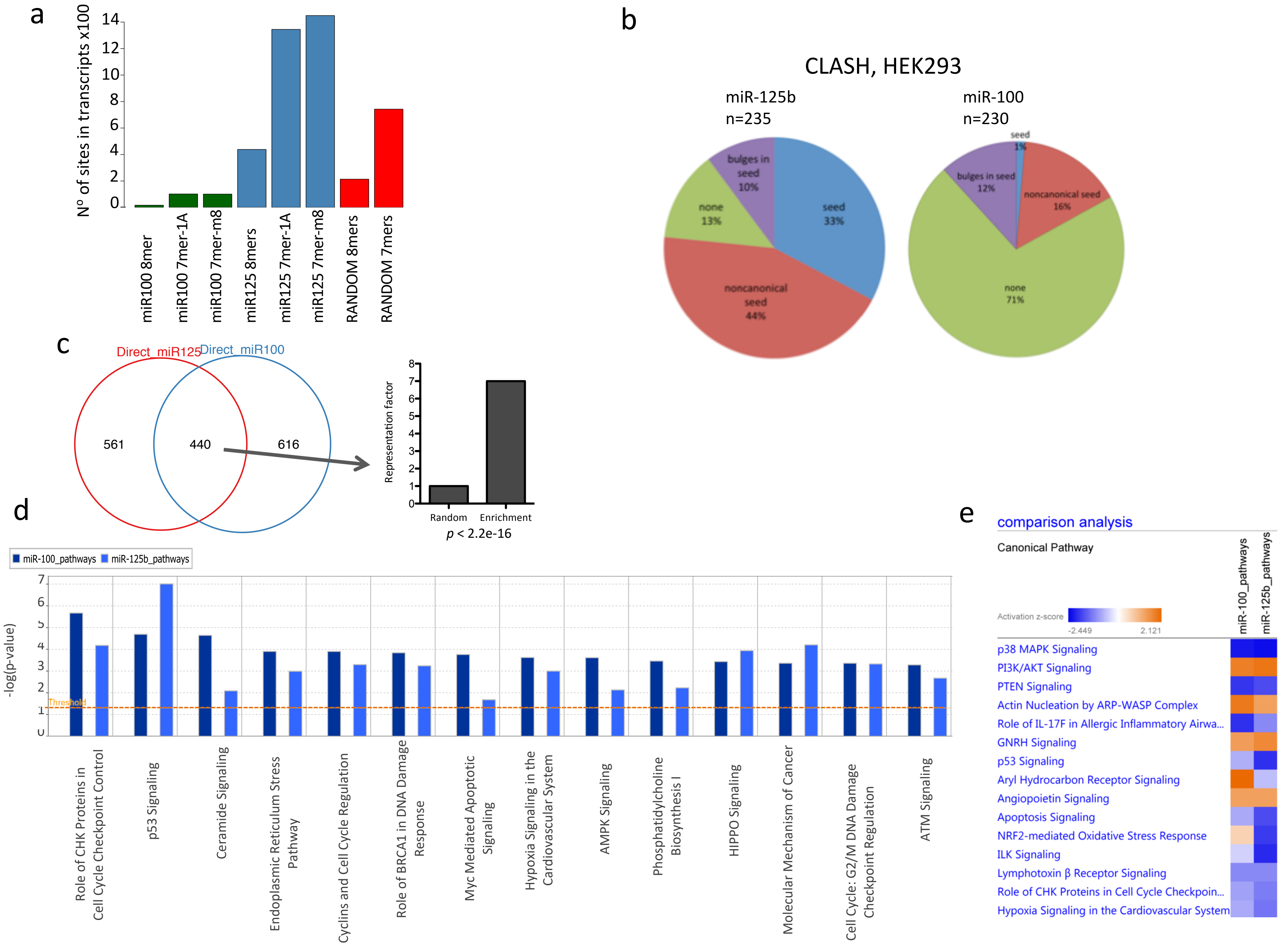
a
N° of sites in transcripts x100
miR100 8mer
miR100 7mer-1A
miR100 7mer-m8
miR125 8mers
miR125 7mer-1A
miR125 7mer-m8
RANDOM 8mers
RANDOM 7mers
b
CLASH, HEK293
miR-125b
n=235
miR-100
n=230
seed 33%
bulges in seed 10%
none 13%
noncanonical seed 44%
seed 1%
bulges in seed 12%
noncanonical seed 16%
none 71%
c
Direct_miR125
Direct_miR100
561
440
616
Representation factor
Random
Enrichment
p < 2.2e-16
d
miR-100_pathways
miR-125b_pathways
-log(p-value)
Threshold
Role of CHK Proteins in Cell Cycle Checkpoint Control
p53 Signaling
Ceramide Signaling
Endoplasmic Reticulum Stress Pathway
Cyclins and Cell Cycle Regulation
Role of BRCA1 in DNA Damage Response
Myc Mediated Apoptotic Signaling
Hypoxia Signaling in the Cardiovascular System
AMPK Signaling
Phosphatidylcholine Biosynthesis I
HIPPO Signaling
Molecular Mechanism of Cancer
Cell Cycle: G2/M DNA Damage Checkpoint Regulation
ATM Signaling
e
comparison analysis
Canonical Pathway
Activation z-score
-2.449
2.121
miR-100_pathways
miR-125b_pathways
p38 MAPK Signaling
PI3K/AKT Signaling
PTEN Signaling
Actin Nucleation by ARP-WASP Complex
Role of IL-17F in Allergic Inflammatory Airwa...
GNRH Signaling
p53 Signaling
Aryl Hydrocarbon Receptor Signaling
Angiopoietin Signaling
Apoptosis Signaling
NRF2-mediated Oxidative Stress Response
ILK Signaling
Lymphotoxin β Receptor Signaling
Role of CHK Proteins in Cell Cycle Checkpoin...
Hypoxia Signaling in the Cardiovascular System

Supplementary Figure 11. miR-100 and miR-125b regulate common genes and pathways**.** (**a**) Barplot showing the number of 8mer, 7mer-1A and 7mer-m8 seeds complementary to miR-100 and miR-125b 5'regions compared to the mean of randomly generated 8mers and 7mers present in human transcripts. (**b**) Pie charts showing the percentage of canonical, noncanonical and unexplained interactions of miR-125b or miR-100 with their targets analyzing the target part derived from miR-125b-target or miR-100-target chimeras from the published CLASH study by Helwak et al. (**c**) Venn diagram showing the significant overlap between miR-125b direct targets (*n*=1001. See also **Fig. 7a**) and miR-100 direct targets (*n*=1056. See also **Fig. 7b**). The bar chart indicates the statistical significance of the overlap between the two groups. (**d-e**) Comparison analysis performed with IPA. Differentially expressed genes (Z-score < -1.5 and Z-score > 1.5) from RNA-seq data of miR-100 or miR-125b overexpression were first analyzed using IPA independently and subsequently merged together for comparison analyses. Comparisons between the two analyses show that both miRNAs activate or inhibit common pathways. In **d** the bar chart shows the most significance pathways from both analyses. In **e** the Heatmap shows activated or inhibited pathways based on IPA Z-score values.

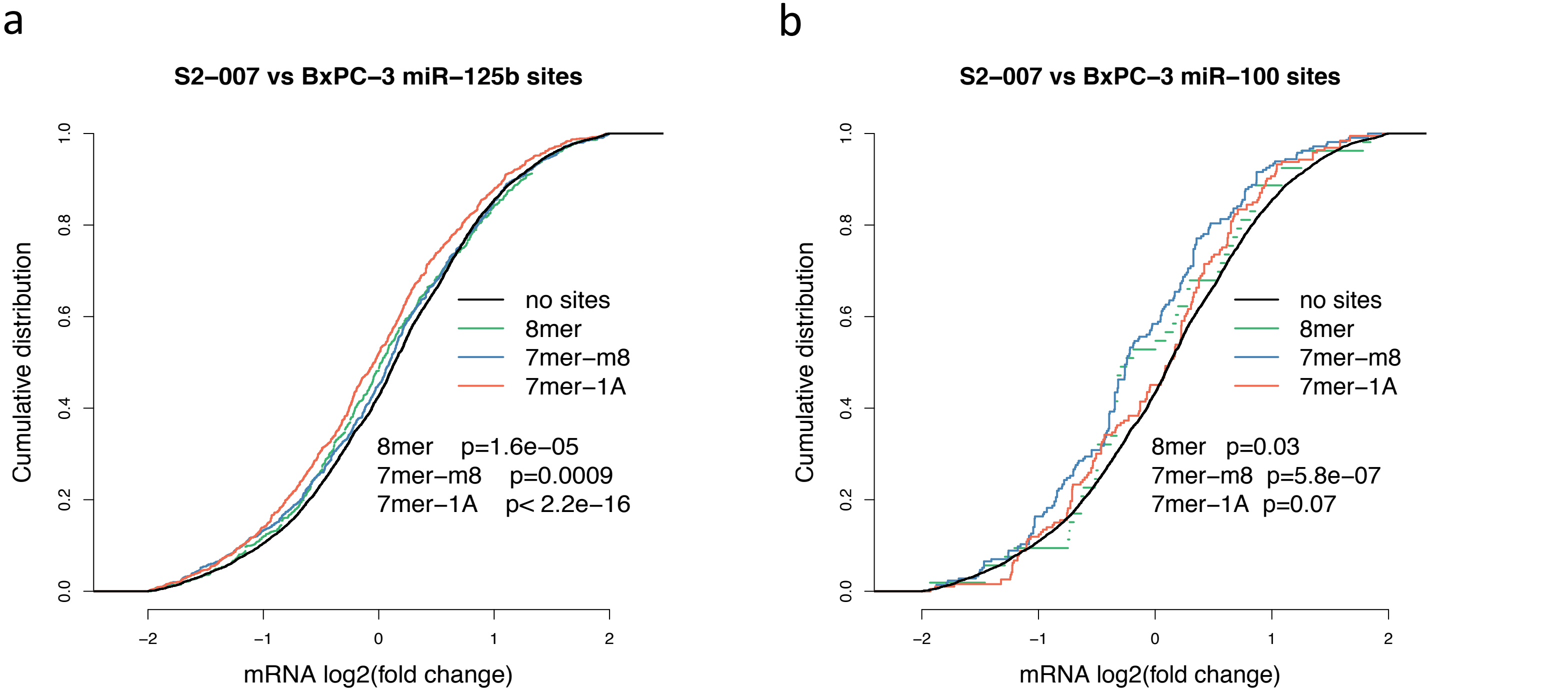

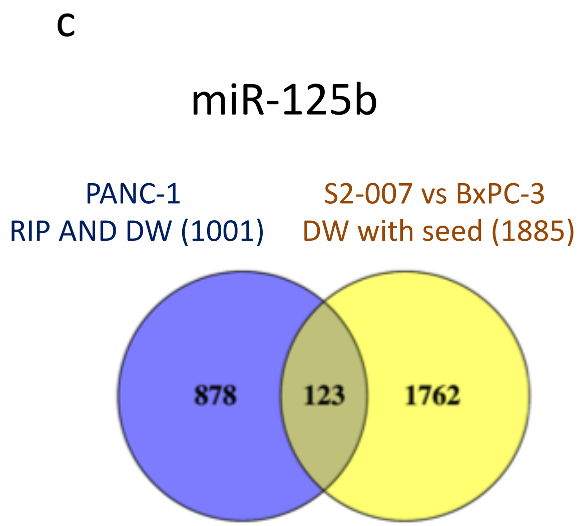

d

Ingenuity Canonical Pathways	-log(p-value)
**Apoptosis Signaling**	2.8
**Integrin Signaling**	2.14
VEGF Family Ligand-Receptor Interactions	1.86
PDGF Signaling	1.83
**Tight Junction Signaling**	1.83
**FAK Signaling**	1.74
VEGF Signaling	1.71
**Transcriptional Regulatory Network in Embryonic Stem Cells**	1.66
AMPK Signaling	1.65
**p53 Signaling**	1.6
ERK/MAPK Signaling	1.58
**Role of Oct4 in Mammalian Embryonic Stem Cell Pluripotency**	1.57

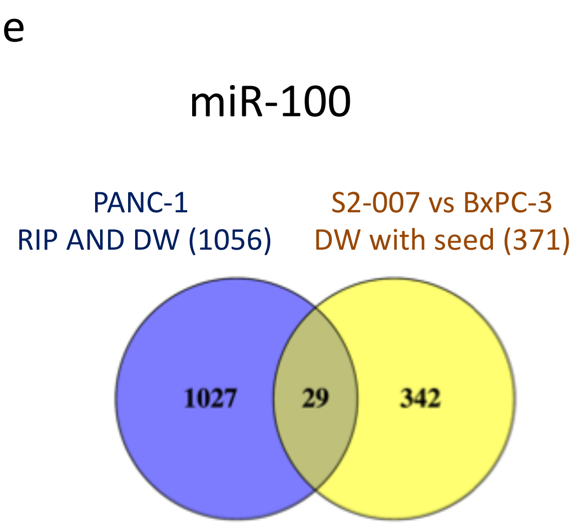

Supplementary Figure 12. Transcripts down-regulated in S2-007 cells expressing high levels of miR-125b and miR-100 versus BxPC-3 are enriched of their seed matches. (**a**) The fold change of transcript levels in S2-007 versus BxPC-3 cells is analyzed comparing transcripts having miR-125b 6-8mer seeds versus transcript lacking them in 3′UTRs. (**b**) The fold change of transcript levels in S2-007 versus BxPC-3 cells is analyzed comparing transcripts having miR-100 6-8mer seeds versus transcript lacking them in 3′UTRs. In **a** and **b** *P*-values were calculated by one-sided Kolmogorov–Smirnov test. (**c**) Venn-diagram showing the overlap of the miR-125b targets identified by RIP-USE in PANC-1 cells and the transcripts down-regulated in S2-007 versus BxPC-3 cells that contain miR-125b seed matches in their 3′UTRs (123). (**d**) IPA pathway analysis of the 123 transcripts belonging to the overlap mentioned in **c**. The relevant pathways for PDAC tumorigenesis and progression are shown in green. (**e**) Venn-diagram showing the overlap of the miR-100 targets identified by RIP-USE in PANC-1 cells and the transcripts down-regulated in S2-007 versus BxPC-3 cells that contain miR-100 seed matches in their 3′UTRs.

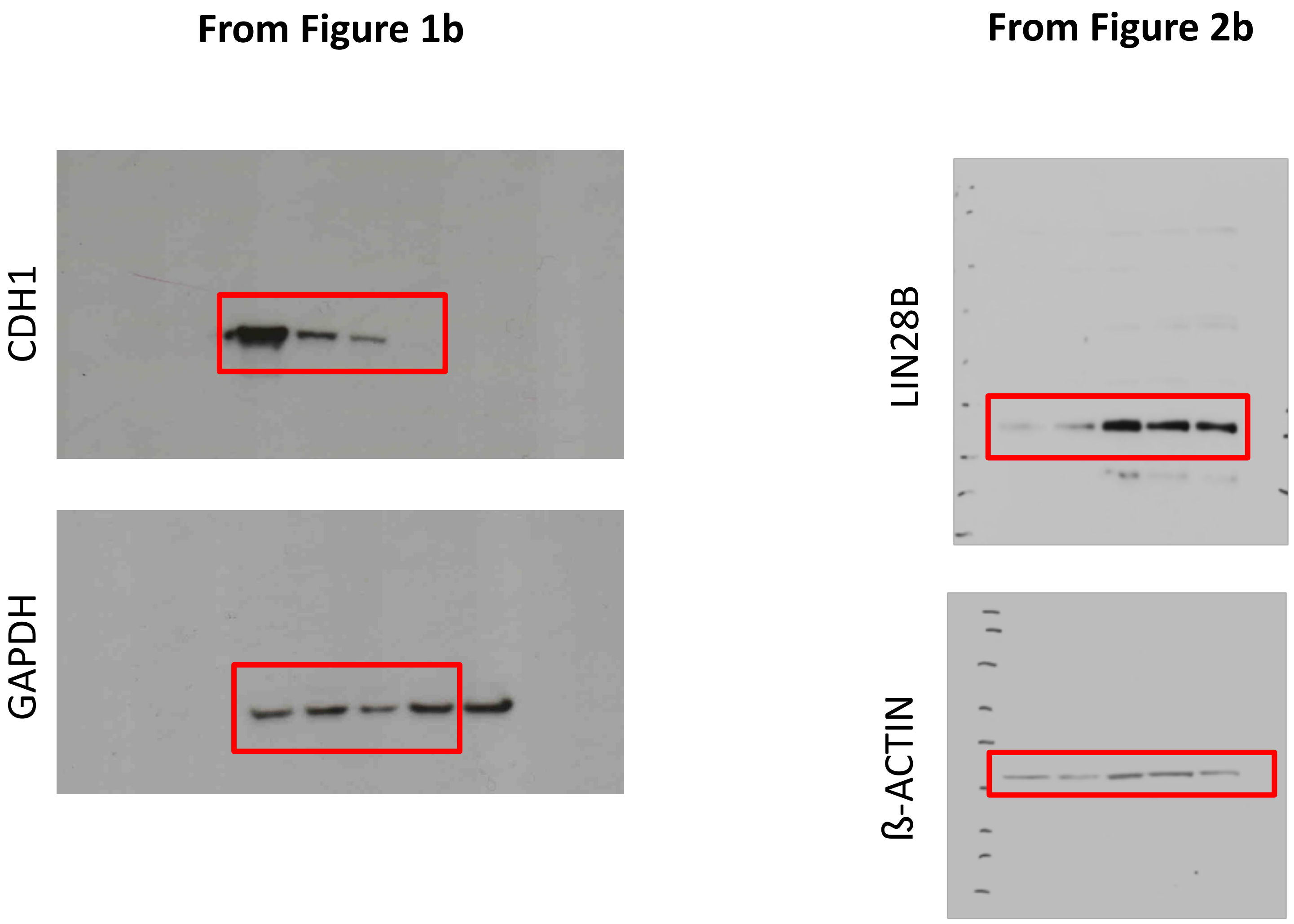

Supplementary Figure 13. Uncropped western blots from main figures. Shown are uncropped blots from figure 1b and 2b. The cropped region is highlighted with the red boxes.

Supplemental Table 1.						
miRNA	Direction of expression (PDAC vs. Normal)	PDAC (n)	Normal (n)	Fold-change (PDAC vs. Normal)	P value	Reference
**miR-100**	Up	8	5	1.18	2.23E-03	1
	Up	17	17	2.27	0.0450485	2
	Up	136	22	1.85	1.50257E-05	3
	Up	28	15	5.00	0.000126041	4
	Up	65	65	2.49	<0.05	5
	Up	48	10	2.83	0.000301	6
	Up	17	15	n/a	0.04	7
	Up	5	5	2.95	<0.001	8
	Up	9	9	n/a	<0.05	9
	Up	10	10	3.29	<0.05	10
**Summary**	**Up-regulated miR-100**	**343**	**173**			
**miR-125b**	Up	8	5	1.12	3.07E-03	1
	Up	136	22	2.50	1.22644E-09	3
	Up	28	15	2.61	0.004962441	4
	Up	65	65	2.03	<0.05	5
	Up	9	9	n/a	<0.05	9
	Up	10	10	3.16	<0.05	10
	Up	3	3	n/a	<0.05	11
**Summary**	**Up-regulated miR-125b**	**258**	**129**			

Supplementary Table 1. Data mining reveals miR-100 and miR-125b are up-regulated in PDAC in several independent cohorts. References for the table: **1**. Szafranska, A. E. *et al.* MicroRNA expression alterations are linked to tumorigenesis and non-neoplastic processes in pancreatic ductal adenocarcinoma. *Oncogene* 26, 4442-4452, (2007). **2**. Piepoli, A. *et al.* Mirna Expression Profiles Identify Drivers in Colorectal and Pancreatic Cancers. *PLoS One* 7, e33663, (2012). **3**. Bauer, A. S. *et al.* Diagnosis of pancreatic ductal adenocarcinoma and chronic pancreatitis by measurement of microRNA abundance in blood and tissue. *PLoS One* 7, e34151, (2012). **4**. Lee, E. J. *et al.* Expression profiling identifies microRNA signature in pancreatic cancer. *International journal of cancer.* 120, 1046-1054, (2007). **5**. Bloomston, M. *et al.* MicroRNA expression patterns to differentiate pancreatic adenocarcinoma from normal pancreas and chronic pancreatitis. *JAMA* 297, 1901-1908, (2007). **6**. Jamieson, N. B. *et al.* MicroRNA Molecular Profiles Associated with Diagnosis, Clinicopathologic Criteria, and Overall Survival in Patients with Resectable Pancreatic Ductal Adenocarcinoma. *Clinical Cancer Research* 18, 534-545, (2012). **7**. Panarelli, N. C., Chen, Y. T., Zhou, X. K., Kitabayashi, N. & Yantiss, R. K. MicroRNA expression aids the preoperative diagnosis of pancreatic ductal adenocarcinoma. *Pancreas* 41, 685-690, (2012). **8**. Nagao, Y. *et al.* Association of microRNA-21 expression with its targets, PDCD4 and TIMP3, in pancreatic ductal adenocarcinoma. *Mod Pathol* 25, 112-121, (2012). **9**. Frampton, A. E. *et al.* MicroRNAs Cooperatively Inhibit a Network of Tumor Suppressor Genes to Promote Pancreatic Tumor Growth and Progression. *Gastroenterology* 146, 268-277 e218, (2014). **10**. Zhang, X. J. *et al.* Dysregulation of miR-15a and miR-214 in human pancreatic cancer. *Journal of hematology & oncology* 3, 46, (2010). **11**. Ma, Y., Yu, S., Zhao, W., Lu, Z. & Chen, J. miR-27a regulates the growth, colony formation and migration of pancreatic cancer cells by targeting Sprouty2. *Cancer Lett*, (2010).

Supplementary Table 2.							
**Variable**	**All cases n=100 (%)**	**Low miR-100 n (%)**	**High miR-100 n (%)**	**X^2 (2-tailed) P values**	**Low miR-125b n (%)**	**High miR-125b n (%)**	**X^2 (2-tailed) P values**
		52 (52)	48 (48)		52 (52)	48 (48)	
**Differentiation grade**[a]							
**Low**	72 (72)	39 (54.2)	33 (45.8)	0.487	42 (58.3)	30 (41.7)	**0.042**[b]
**High**	28 (28)	13 (46.4)	15 (53.6)		10 (35.7)	18 (64.3)	

MiRNA expression was defined as high or low in terms of above or below the mean miRNA ISH score respectively.[a]High grade tumors were poorly differentiated, compared to low grade which were moderately or well differentiated; [b]Statistically significant.

Supplementary Table 2. Correlation between miR-100 and miR-125b tumoral expression with grade of differentiation (n=100)**.** High grade tumors were associated with high miR-125b expression (*P*=0.042), but not higher miR-100 expression.
