# Supplementary material for: TGF-β induces miR-100 and miR-125b but blocks let-7a through LIN28B controlling PDAC progression
Source: Nat Commun. 2018 May 10;9:1845. doi: 10.1038/s41467-018-03962-x (PMC5945639; doi:10.1038/s41467-018-03962-x)
Supplement: Supplementary file 3 — Description of Additional Supplementary Files [file 41467_2018_3962_MOESM3_ESM.pdf]

Supplementary Data 1.

nCounter miRNA expression profiling in pancreatic cancer cell lines.

Supplementary Data 2.

DESeq2 analysis of RNA-seq from PANC-1 treated with TGF-ß.

Supplementary Data 3.

RIP analysis from miR-100 or miR-125b overexpression in PANC-1 cells.

Supplementary Data 4.

List of differentially expressed genes from miR-100 or miR-125b overexpression in PANC-1 cells.

Supplementary Data 5.

miR-125b or miR-100 target networks.

Supplementary Data 6.

Comparison analysis between miR-100 and miR-125b most significant pathways.

Supplementary Data 7.

Normalized levels of miRNAs in BxPC-3 and S2-007 from nCounter miRNA expression profiling (miRNAs are ranked from the most to the least expressed in S2-007 cells).

Supplementary Data 8.

DESseq2 analysis of RNA-seq count values identifies differentially expressed genes in S2-007 versus BxPC-3 cells.

Supplementary Movie 1.

Time-lapse microscopy wound healing assay in S2-007 Zc-11 cells.

Supplementary Movie 2.

Time-lapse microscopy wound healing assay in S2-007 Z100-3 cells.

Supplementary Movie 3.

Time-lapse microscopy wound healing assay in S2-007 Z125b-5 cells.
